# Post-diagnostic health behaviour scores in relation to fatal prostate cancer

**DOI:** 10.1038/s41416-022-01948-7

**Published:** 2022-08-26

**Authors:** Rebecca E. Graff, Crystal S. Langlais, Erin L. Van Blarigan, Claire H. Pernar, Meir J. Stampfer, Edward L. Giovannucci, Lorelei A. Mucci, June M. Chan, Stacey A. Kenfield

**Affiliations:** 1 Department of Epidemiology and Biostatistics, University of California, San Francisco, San Francisco, CA USA; 2 Department of Epidemiology, Harvard T.H. Chan School of Public Health, Boston, MA USA; 3 Department of Urology, University of California, San Francisco, San Francisco, CA USA; 4 Department of Nutrition, Harvard T.H. Chan School of Public Health, Boston, MA USA; 5 Channing Division of Network Medicine, Department of Medicine, Brigham and Women’s Hospital and Harvard Medical School, Boston, MA USA

**Keywords:** Prostate cancer, Risk factors, Epidemiology

## Abstract

**Background:**

Individual health behaviours have been associated with fatal prostate cancer (PCa). Their combined association with fatal PCa *after* diagnosis is unknown.

**Methods:**

This prospective cohort included 4518 men diagnosed with nonmetastatic PCa from the Health Professionals Follow-up Study. Exposures included a three-factor score integrating post-diagnostic fatal PCa risk factors (“2021 PCa Behaviour Score”), six-factor score integrating incident aggressive PCa risk factors (“2015 PCa Behaviour Score”), and two scores integrating recommendations for cancer prevention and survival, respectively. Multivariable Cox models estimated hazard ratios (HRs) and 95% confidence intervals (CIs) for fatal PCa.

**Results:**

Over a median 10.2 years, we observed 219 PCa deaths. Each additional point of one of the PCa-specific health behaviour scores (2015 PCa Behaviour Score) was associated with a 19% reduced fatal PCa risk (HR: 0.81, 95%CI: 0.68–0.97). The 2021 PCa Behaviour Score and scores integrating national recommendations were not associated with fatal PCa.

**Conclusions:**

While a PCa-specific health behaviour score was associated with a reduced risk of fatal PCa, we did not otherwise observe strong evidence of associations between post-diagnostic scores and fatal PCa. Avoiding tobacco, healthy body size, and physical activity may decrease PCa death risk, but further research is needed to inform cancer survivorship recommendations.

## Background

Over 3.1 million men live with diagnosed prostate cancer (PCa) in the United States, and >190,000 new cases are diagnosed each year [1, 2]. Disease progression is a pivotal concern among patients with nonmetastatic PCa, so identifying ways to lower progression risk is an important public health need. Individual health behaviours (e.g., smoking, exercise, dietary factors) have been associated with the risk of fatal PCa, but no observational studies have examined them in combination after diagnosis in relation to PCa death.

In 2015, we developed a six-factor health behaviour score for the prevention of lethal (metastatic plus fatal) PCa among healthy men in the Health Professionals Follow-up Study (HPFS; “2015 PCa Behaviour Score”). We reported that men with 5–6 vs. 0–1 points (i.e., healthier vs. less healthy behaviours) had a 68% decreased risk of developing lethal PCa [3], and we found similar results when evaluating the score in the Physicians’ Health Study [3]. To date, no score combining health behaviours *after* diagnosis has been developed for the prevention of fatal PCa. Given that a cancer diagnosis represents a potential “teachable moment” [4–6], it is important to understand the combination of post-diagnostic health behaviours that best precludes disease progression. We thus reviewed the evidence examining individual health behaviours after PCa diagnosis [7] and developed a novel, literature-based, post-diagnostic health behaviour score comprised of factors that have been associated with fatal outcomes (“2021 PCa Behaviour Score”; see “Methods”).

The World Cancer Research Fund (WCRF)/American Institute for Cancer Research (AICR) and American Cancer Society (ACS) developed diet and exercise guidelines for cancer prevention (to be followed by cancer survivors, if feasible; “WCRF/AICR Score”) [8] and cancer survivors (“ACS Score”) [9], respectively. Shams-White, et al. operationalized the WCRF/AICR recommendations [10, 11], and investigators have operationalized the ACS guidelines to analyse them with respect to cancer-specific survival and all-cause and cause-specific death [12, 13]. Whether practices consistent with these recommendations are associated with better outcomes among men with PCa is unknown.

To comprehensively evaluate whether combined behaviours after diagnosis are associated with the risk of fatal PCa, we examined all four aforementioned scores: the 2015 score based on pre-diagnostic behaviours, the 2021 score based on the post-diagnostic behaviour literature, and the two national organisation scores. We leveraged the HPFS, which includes a large cohort of men diagnosed with PCa who have reported health behaviour data every 2–4 years (including before and after diagnosis), as well as cancer diagnostic and treatment information. We hypothesised that higher scores (reflecting greater alignment with health recommendations) would be associated with lower risk of poor outcomes.

## Methods

### Study population

The HPFS is an ongoing prospective cohort of 51,529 male health professionals who were ages 40–75 at enrollment in 1986. Participants responded to a baseline questionnaire concerning medical history, medications, lifestyle, and diet, and have since completed follow-up questionnaires biennially. Average follow-up rates exceed 90%.

**Fig. 1:**
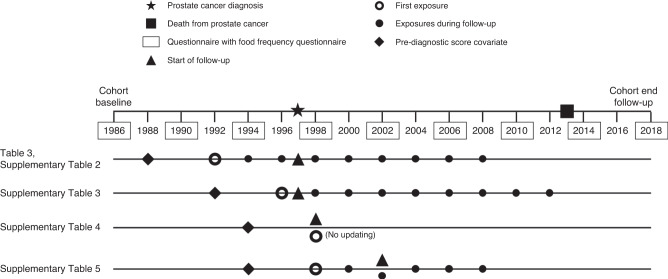
Schematic of follow-up, exposure, and covariate timing for the primary and sensitivity analyses. The analyses are depicted for a hypothetical individual diagnosed in 1997, who died from prostate cancer in 2013. Dietary information in years lacking a food frequency questionnaire was carried forward from prior years. The exception was the transition from pre- to post-diagnosis; if the first questionnaire administered after diagnosis did not include a food frequency questionnaire, then dietary data were considered missing. The analyses corresponding to Table 3 and Supplementary Table 2 started follow-up at the time of diagnosis and lagged exposure by 4–6 years. The analyses corresponding to Supplementary Table 3 started follow-up at the time of diagnosis and did not lag exposure. The analyses corresponding to Supplementary Table 4 started follow-up at the time of first post-diagnosis questionnaire with all score components and anchored exposure at first post-diagnostic health behaviour scores without updating. The analyses corresponding to Supplementary Table 5 started follow-up 4–6 years after the first post-diagnosis questionnaire with all score components and lagged exposure 4–6 years.

Primary analyses were restricted to individuals diagnosed with PCa after return of the 1994 follow-up questionnaire (*n* = 5695) so that we were able to address reverse causation by lagging exposure 4–6 years (beginning in 1990) and address confounding by adjusting for behaviours 8–10 years prior to diagnosis (beginning in 1986) (Fig. 1). (In the first post-diagnostic follow-up windows, exposure was measured in the years leading up to diagnosis.) We excluded 333 individuals diagnosed with any cancer (other than non-melanoma skin cancer) prior to 1994 and 17 individuals known to have died but whose date of death was unavailable. Among the remaining participants with PCa, we excluded 166 diagnosed with T3b or higher disease and 597 with unknown stage at diagnosis, such that the remaining 4582 men were diagnosed with stage T3a or lower disease. We further restricted analyses of each score to individuals with values of the score 4–6 years and 8–10 years prior to diagnosis. The number excluded due to missing data was specific to each score based on participants having responded to questionnaire items corresponding to score components (2021 PCa Behaviour Score: 220; 2015 PCa Behaviour Score: 262; WCRF/AICR Score: 64; ACS Score: 77).

### Smoking, anthropometry, and physical activity assessment

Current smoking status was assessed on all biennial questionnaires. In addition, the 1986 questionnaire inquired about past smoking behaviour and, for past smokers, time since quitting. At every questionnaire cycle, we classified participants as current smoker, quit ≥10 years prior, quit <10 years prior, or never-smoker. Body mass index (BMI; kg/m^2^) was determined at each questionnaire cycle by dividing current weight by the square of height reported in 1986. Physical activity was derived using a validated survey that inquired about time spent participating in a variety of leisure-time activities (e.g., walking, jogging, bicycling, etc.) [14]. Participants indicated low, medium, or high intensity of bicycling, swimming, and tennis starting in 2010; intensity before then and for other activities was considered to be medium. To calculate metabolic equivalent task (MET) hours per week, we assigned each activity a MET value, multiplied the value by the amount of time spent engaging in the activity, and summed across activities.

### Diet assessment

In 1986 and every 4 years thereafter, participants completed a validated, semiquantitative food frequency questionnaire (FFQ) [15, 16]. For each food or drink item, a commonly used unit or portion size was specified, and participants were asked how often, on average, over the past year they had consumed that amount of each item. Participants could choose from nine possible frequencies ranging from never to six or more times per day. The validity of FFQs relative to diet records in the HPFS has been previously reported [15].

To minimise random within-person variation and to best represent long-term post-diagnostic intake [12], we used cumulative average intakes of total energy, nutrients, foods, and beverages in the scores and as covariates. For example, for an individual diagnosed in 1997, we used the 1998 FFQ to define first post-diagnostic dietary values. For the same individual, second post-diagnostic values were calculated by averaging intakes from the 1998 and 2002 FFQs, and so forth. For pre-diagnostic diet, we used cumulative average intakes from baseline through the last pre-diagnostic FFQ (e.g., the last pre-diagnostic value for the example individual was calculated by averaging intakes from the 1986, 1990, and 1994 FFQs).

### Development of lifestyle scores

The individual items included in the score components are shown in Supplementary Table 1. Additional details pertaining to the development and operationalization of each score are described below. All scores were constructed such that higher scores corresponded to healthier behaviours.

#### 2015 PCa behaviour score

In 2015, our group created a health behaviour score for the risk of developing lethal PCa in cancer-free men [3]. Six factors—smoking status, BMI, physical activity, processed red meat intake, tomato intake, and fatty fish intake—were dichotomised based on standard definitions or previously reported cut-points (Table 1) [17–22]. Men were assigned one point for each factor (score range: 0–6). We also performed analyses excluding smoking status from the 2015 PCa Behaviour Score (“2015 PCa Behaviour Score Excluding Smoking Status”; score range: 0–5; results in Supplementary Table 2). While it was developed in the context of PCa risk, here we evaluated the association between the 2015 PCa Behaviour Scores and outcomes among men already diagnosed with PCa.

**Table 1:** Components and point assignment of the 2021 PCa Behaviour Score, 2015 PCa Behaviour Score, WCRF/AICR Score, and ACS Score^a^.

*ACS* American Cancer Society, *AICR* American Institute for Cancer Research, *aUPF* adapted ultra-processed food, *IQR* interquartile range, *MET* metabolic equivalent of task, *PCa* prostate cancer, *serv* servings, *WCRF* World Cancer Research Fund.

^a^Score components bordered by bold boxes were combined together into single subscores.

^b^Median (IQR) at the time of first available post-diagnostic questionnaire with all score components.

^c^Only the secondary version of the 2021 PCa Behaviour Score (point range 0–4) included the percentage of calories from saturated fat, whole milk, wine, and processed meat, the four of which were summed and divided by four.

^d^Fibre and whole fruits and vegetables were summed and then divided by two.

^e^Dietary factors—fruit and vegetable servings, fruit and vegetable variety, red and processed meat, and percentage of grains that are whole—were summed, and individuals with 0–2 points were given 0 overall diet points, individuals with 3–6 points were given 1 overall diet point, and individuals with 7–9 points were given 2 overall diet points.

^f^Only the secondary version of the ACS Score (point range 0–8) included alcohol.

^g^The 2021 PCa Behaviour and ACS Scores excluded individuals with BMI < 18.5 kg/m^2^.

^h^Hours per week of vigorous physical activity were doubled in the calculation for overall hours per week of moderate or vigorous physical activity.

#### 2021 PCa Behaviour Score

We developed the novel 2021 PCa Behaviour Score based on literature regarding post-diagnostic factors and the risk of PCa-specific death [7]. Factors considered for the score: (1) exhibited a statistically significant association in at least one study; and (2) were corroborated by at least one additional study with an association in the same direction, whether or not statistically significant. In total, we identified seven such factors—smoking status [23–32], BMI [33–49], physical activity [50–54], and intake of saturated fat [55–57], whole milk [58, 59], wine [60, 61], and processed meat [62, 63]. The primary 2021 PCa Behaviour Score included only smoking status, BMI, and physical activity, as these three factors demonstrated the strongest evidence of associations with outcomes and are more likely than dietary factors to be collected in other studies and the clinic. We established cut-points for each score component according to standard guidelines or based on reviewing prior reports and observing levels at which the risk of outcomes appeared to change (Table 1). Smoking status, BMI, and physical activity were scaled from zero to one, with the allowance of half-point values for partial adherence to healthy behaviours (2021 PCa Behaviour Score range: 0–3).

A secondary version of the score added the abovementioned dietary factors to smoking status, BMI, and physical activity (“2021 PCa Behaviour Score Including Diet”; score composition in Table 1, results in Supplementary Table 2). Dietary factors were assigned a value of zero or one, after which they were summed and divided by four; up to one point could thus be assigned for dietary factors (2021 PCa Behaviour Score Including Diet range: 0–4). To facilitate clearer comparisons with the WCRF/AICR and ACS Scores, we also conducted a sensitivity analysis in which we evaluated the primary score (i.e., excluding diet) without the smoking status component (“2021 PCa Behaviour Score Excluding Smoking Status”; score range: 0–2; results in Supplementary Table 2).

#### WCRF/AICR Score

We based our operationalization of the 2018 WCRF/AICR Cancer Prevention Recommendations [8] on a published standardised scoring system [10, 11]. Each of seven score components—BMI, physical activity, alcohol intake, red and processed meat intake, fruit and vegetable/fibre intake, sugar-sweetened beverage intake, and percentage of total calories from ultra-processed foods (excluding foods already in the score)—was scored from zero to one, allowing for partial adherence (Table 1; score range: 0–7).

#### ACS Score

Adherence to the American Cancer Society Nutrition and Physical Activity Guidelines for Cancer Survivors [9] was quantified according to the principles of McCullough et al. [12]. To be consistent with the guidelines, however, we included strength training when assigning points for physical activity. First, we created a dietary sub-score comprised of red and processed meat intake, percentage of grains consumed that are whole, fruit and vegetable intake, and variety of fruits and vegetables consumed. The dietary sub-score and BMI and physical activity components were each scaled from 0 to 2 (Table 1; score range: 0–6). Because the ACS addresses alcohol intake in its guidelines for cancer prevention but not for cancer survival, and based on established precedent [12], we also created a secondary version of the ACS Score that included alcohol intake (“ACS Score Including Alcohol”; score range: 0–8; results in Supplementary Table 2).

### Ascertainment of PCa diagnoses and outcomes

PCa cases were initially identified via self-report or participants’ next-of-kin and confirmed by medical record and pathology report. Study investigators reviewed records and PCa-specific follow-up questionnaires to abstract information about the clinical stage, Gleason score, prostate-specific antigen (PSA) levels, treatments, and disease progression over time. Deaths were ascertained via reports from family members, the postal system, or the National Death Index. Follow-up for mortality was over 98% complete. Study physicians reviewed medical records and death certificates to determine cause of death, including from PCa, where applicable. Cases were defined as fatal if they died from PCa.

### Statistical analysis

These analyses focused on post-diagnostic health behaviours but included pre-diagnostic values of the scores as exposures in early follow-up periods of the primary lagged analyses and as covariables across all analyses (Fig. 1). When health behaviour score components were missing, we carried forward values within the pre- and post-diagnostic periods. Exposure data were not carried forward from pre- to post-diagnosis. All lifestyle scores were modelled both continuously and categorically. For the latter, each score was split into five categories, with ~10% of participants in each of the extreme categories and the remaining participants distributed across the three middle categories. The 2015 PCa Behaviour Score was categorised as previously published [3]. The distribution of the 2015 PCa Behaviour Score Excluding Smoking Status allowed for only four categories.

For our primary analyses, person-time was calculated from the date of diagnosis until date of death or end of follow-up on January 1, 2017. In preliminary age-adjusted analyses, we then used time-dependent Cox proportional hazards models stratified on calendar year and years since diagnosis to estimate hazard ratios (HRs) and 95% confidence intervals (CIs) for each post-diagnostic score and fatal PCa. Exposure in the first follow-up period was assigned as the score 4–6 years prior to diagnosis; exposure was then updated every questionnaire cycle throughout the post-diagnostic period. We also ran multivariable models additionally adjusted for non-Hispanic White (hereafter “White”) race/ethnicity (yes, no); family history of PCa in a father or brother (yes, no); supplemental selenium use (non-user, <140 μg daily, ≥140 μg daily); daily energy intake (continuous); and the following covariates if they were not in the exposure score (e.g., models of the 2021 PCa Behaviour Score were not adjusted for smoking): smoking status (never, past-quit ≥10 years prior, past-quit <10 years prior, current); whole milk intake (>4 servings/week, ≤4 servings/week); wine intake (3–14 servings/week, <3 or >14 servings/week); total alcohol intake (non-drinker, >0–2 servings/day, >2 servings/day); processed meat intake (quartiles); tomato intake (continuous); and/or fatty fish intake (continuous). Further models additionally adjusted for clinical characteristics, including clinical stage (T1, T2, T3a), Gleason score (<7, 7, >7, missing), diagnostic PSA level (≤6 ng/mL, >6–10 ng/mL, >10–20 ng/mL, >20 ng/mL, missing), and primary treatment (radical prostatectomy, radiation, hormonal therapy, watchful waiting, other, missing). Our final multivariable models also adjusted for pre-diagnostic values of the relevant health behaviour score. We examined models additionally adjusted for multivitamin use (never, past, current), choline intake (continuous), coffee intake (continuous), vegetable fat intake (continuous; 2021 PCa Behaviour Score, 2021 PCa Behaviour Score Including Diet, and 2015 PCa Behaviour Score), hypertension (yes, no), elevated cholesterol (yes, no), and comorbidities (at least one comorbidity, no comorbidities). Results were materially unchanged, so these variables were omitted.

To explore effect modification, we stratified models by age at diagnosis (<65 years, ≥65 years) and, separately, stage at diagnosis (T1, T2/T3a). Interaction with age and stage was assessed by Wald tests for interaction terms.

In sensitivity analyses that included PCa diagnoses beginning in 1990, we restricted to individuals with values of each score immediately prior to diagnosis (beginning in 1990) and 4–6 years prior (beginning in 1986) (results in Supplementary Table 3). In each period of these unlagged analyses, exposure corresponded to the most recent questionnaire (i.e., rather than the questionnaire administered 4–6 years prior). These analyses considered more acute impacts of health behaviours on PCa outcomes but were more susceptible to reverse causation. Sample sizes were larger than those in the primary analyses (2021 PCa Behaviour Score: 5335; 2015 PCa Behaviour Score: 5274; WCRF/AICR Score: 5502; ACS Score: 5486).

We also ran sensitivity analyses including diagnoses from 1988 to 2016 that initiated follow-up on the date of return of the first post-diagnostic questionnaire with data on all relevant score components and did not update exposure after baseline (2021 PCa Behaviour Score: 4968; 2015 PCa Behaviour Score: 4605; WCRF/AICR Score: 5223; ACS Score: 5208) (results in Supplementary Table 4). Anchoring exposure at the first available post-diagnostic questionnaire limits reverse causation and allows for comparisons with cohorts that do not collect health behaviours longitudinally but may miss relevant aetiologic windows of exposure.

In final sensitivity analyses including diagnoses from 1988 to 2012, we maintained a 4–6 year lag, excluding post-diagnostic periods for which lagged exposure was pre-diagnostic (2021 PCa Behaviour Score: 4559; 2015 PCa Behaviour Score: 3900; WCRF/AICR Score: 4375; ACS Score: 4363) (results in Supplementary Table 5). For example, for an individual diagnosed in 1989, scores from the 1990 questionnaires were used to assign exposure in the first follow-up period from 1994 to 1996. In contrast to our primary analyses that included 4–6 years of pre-diagnostic exposure applied to the first post-diagnostic follow-up windows, these analyses captured exclusively post-diagnostic behaviours. Though vulnerable to survival bias (since participants had to survive at least 4–6 years after diagnosis to be included), they addressed reverse causation and considered only post-diagnostic exposure.

We verified the proportional hazards assumption via likelihood ratio tests comparing the primary fully adjusted models with and without interactions between time scale and exposure. All statistical analyses were performed using SAS version 9.3 (SAS Institute, Cary, NC). Two-sided *P* values <0.05 were considered statistically significant.

## Results

Characteristics of men in extreme categories of each of the four main scores according to the first available post-diagnostic questionnaire are presented in Table 2. Higher values of the scores were generally associated with lower PSA values at diagnosis, as well as primary treatment with radical prostatectomy. Distributions of health behaviours corresponding to each score are shown in Supplementary Table 6. Spearman correlations between the first available post-diagnostic score and the last pre-diagnostic score at least 4 years prior were 0.62, 0.54, 0.56, and 0.66 for the 2021 PCa Behaviour Score, 2015 PCa Behaviour Score, WCRF/AICR Score, and ACS Score, respectively.

**Table 2:** Age-adjusted characteristics of Health Professionals Follow-up Study participants with nonmetastatic PCa, according to extreme categories of first health behaviour scores following diagnosis.

Sample size:	2021 PCa Behaviour Score, *n* = 4968		2015 PCa Behaviour Score, *n* = 4605		WCRF/AICR Score, *n* = 5223		ACS Score, *n* = 5208	
# Points:	0–1	3	0–1	5–6	0–2.5	5.25–7	0–1.5	5–6
*n*	423	1170	173	645	635	609	568	705
Mean age at diagnosis, years ± SD	69 ± 6.7	70 ± 7.0	69 ± 6.7	69 ± 6.8	69 ± 7.4	70 ± 6.7	70 ± 6.7	69 ± 6.8
Age at diagnosis, %								
<65	30	23	25	28	26	22	23	26
65–69	28	26	35	26	28	29	30	26
70–74	22	26	24	27	23	27	26	27
75–79	15	18	13	14	15	16	15	15
≥80	5.2	6.8	2.9	5.0	7.4	6.2	6.0	5.3
Stage at diagnosis, %								
T1/N0/M0	57	61	58	66	65	57	62	63
T2/N0/M0	42	37	41	31	34	40	36	35
T3a/N0/M0	1.4	1.9	1.7	2.4	1.5	2.4	2.1	2.0
Gleason score at diagnosis^a^, %								
<7	55	60	60	59	62	59	58	58
7	31	30	26	31	26	31	29	32
>7	14	9.6	15	9.9	11	11	12	10
Median PSA at diagnosis^a^, ng/mL (IQR)	7.0 (4.7, 11)	6.6 (4.8, 10)	7.0 (4.9, 10)	6.0 (4.6, 9.2)	6.5 (4.7, 9.8)	6.4 (4.6, 9.7)	7.0 (4.9, 10)	6.3 (4.7, 9.1)
PSA at diagnosis^a^, %								
≤6	40	43	40	50	46	46	40	46
>6–10	31	32	37	28	31	32	34	33
>10–20	18	19	11	16	16	17	18	15
>20	11	5.7	12	6.2	7.4	6.2	8.3	5.5
Primary treatment^a^, %								
Radical prostatectomy	40	52	43	43	43	48	43	50
Radiation	40	34	43	42	42	35	43	36
Hormone therapy	9.8	4.1	7.3	3.3	4.8	6.0	6.0	3.6
Watchful waiting	7.1	8.1	3.8	9.9	7.5	8.9	6.2	8.7
Other	3.3	1.6	2.5	1.6	2.4	1.7	1.7	1.4
Year of diagnosis, %								
1994 or earlier	23	23	25	17	16	25	18	17
1995–1999	22	21	18	21	24	20	23	21
2000–2004	27	23	29	26	28	25	30	23
2005–2009	21	24	20	25	24	19	20	26
2010 or later	7.5	9.3	6.8	11	7.8	12	8.4	13
Non-Hispanic White, %	95	97	95	97	96	95	97	97
Family history of PCa, %	11	18	18	17	15	17	14	17

Notes: all values calculated based on individuals without missing data; percentages may not add up as expected due to rounding.

*ACS* American Cancer Society, *AICR* American Institute for Cancer Research, *IQR* interquartile range, *PCa* prostate cancer, *PSA* prostate-specific antigen, *SD* standard deviation, *WCRF* World Cancer Research Fund.

^a^Among individuals with information available.

Primary analyses of the 2021 PCa Behaviour Score were based on 4362 men diagnosed with stage T3a or lower PCa, followed for a median (interquartile range) of 10 (6.0, 15) years. There were 219 deaths from PCa. Though the 2021 PCa Behaviour Score was associated with a lower risk of fatal PCa in crude models, the association was attenuated upon adjustment for clinical characteristics and pre-diagnostic behaviours (HR_3 pts vs. 0–1 pts_: 0.84, 95% CI: 0.46–1.52, *P*_trend_: 0.77) (Table 3). The absolute crude rates of fatal PCa per 1000 person-years were 4.1 versus 6.7 for those with 3 points vs. 0–1 points, respectively. The 2021 PCa Behaviour Score Including Diet was highly correlated with the primary 2021 PCa Behaviour Score (*r*^*2*^: 0.94), and results from multivariable models were materially unchanged (HR_3.75–4 pts vs. 0–1.5 pts_: 0.75, 95% CI: 0.35–1.59, *P*_trend_: 0.79) (Supplementary Table 2). So too were results for the 2021 PCa Behaviour Score Excluding Smoking (HR_2 pts vs. 0 pts_: 0.85, 95% CI: 0.44–1.63, *P*_trend_: 0.96) (Supplementary Table 2).

**Table 3:** Health behaviour scores lagged by 4–6 years in relation to risk of fatal PCa for Health Professionals Follow-up Study participants diagnosed with nonmetastatic PCa, with follow-up starting at the time of diagnosis.

	2021 PCa Behaviour Score						
	Per point	0–1 pts	1.5 pts	2 pts	2.5 pts	3 pts	*P*_trend_
No. of events/person-years	219/51,372	25/3717	33/7947	56/11,276	53/15,589	52/12,843	
Event rate per 1000 person-years	4.3	6.7	4.2	5.0	3.4	4.1	
Age-Adj HR (95% CI)	0.88 (0.79, 0.98)	1.00	0.54 (0.32, 0.91)	0.58 (0.36, 0.94)	0.46 (0.28, 0.74)	0.50 (0.31, 0.81)	0.02
^a^MV-Adj 1 HR (95% CI)	0.87 (0.78, 0.98)	1.00	0.54 (0.32, 0.91)	0.58 (0.36, 0.93)	0.45 (0.28, 0.74)	0.48 (0.29, 0.79)	0.02
^b^MV-Adj 2 HR (95% CI)	0.94 (0.84, 1.05)	1.00	0.66 (0.39, 1.12)	0.80 (0.49, 1.31)	0.62 (0.37, 1.01)	0.71 (0.42, 1.17)	0.27
^c^MV-Adj 3 HR (95% CI)	0.98 (0.86, 1.12)	1.00	0.68 (0.39, 1.19)	0.88 (0.52, 1.51)	0.71 (0.41, 1.23)	0.84 (0.46, 1.52)	0.77

	2015 PCa Behaviour Score						
	Per point	0–1 pts	2 pts	3 pts	4 pts	5–6 pts	*P*_trend_
No. of events/person-years	183/45,899	13/1993	43/8005	74/18,324	33/12,504	20/5073	
Event rate per 1000 person-years	4.0	6.5	5.4	4.0	2.6	3.9	
Age-Adj HR (95% CI)	0.78 (0.67, 0.90)	1.00	0.74 (0.40, 1.38)	0.50 (0.28, 0.91)	0.33 (0.17, 0.63)	0.51 (0.25, 1.03)	<0.001
^d^MV-Adj 1 HR (95% CI)	0.78 (0.67, 0.90)	1.00	0.73 (0.39, 1.36)	0.50 (0.27, 0.91)	0.32 (0.17, 0.62)	0.51 (0.25, 1.03)	<0.001
^b^MV-Adj 2 HR (95% CI)	0.82 (0.70, 0.95)	1.00	0.80 (0.42, 1.52)	0.58 (0.32, 1.08)	0.39 (0.20, 0.76)	0.64 (0.31, 1.33)	0.01
^c^MV-Adj 3 HR (95% CI)	0.81 (0.68, 0.97)	1.00	0.99 (0.50, 1.94)	0.71 (0.36, 1.41)	0.45 (0.21, 0.95)	0.68 (0.29, 1.57)	0.02

	WCRF/AICR Score						
	Per point	0–2.5 pts	2.75–3.25 pts	3.5–4.25 pts	4.5–5 pts	5.25–7 pts	*P*_trend_
No. of events/person-years	208/49,669	22/5894	49/10,305	66/19,070	45/9300	26/5100	
Event rate per 1000 person-years	4.2	3.7	4.8	3.5	4.8	5.1	
Age-Adj HR (95% CI)	1.01 (0.90, 1.14)	1.00	1.30 (0.78, 2.15)	0.87 (0.54, 1.42)	1.15 (0.69, 1.92)	1.21 (0.68, 2.14)	0.75
^e^MV-Adj 1 HR (95% CI)	0.99 (0.88, 1.13)	1.00	1.30 (0.78, 2.15)	0.84 (0.52, 1.38)	1.12 (0.67, 1.90)	1.10 (0.61, 1.97)	0.99
^b^MV-Adj 2 HR (95% CI)	1.01 (0.89, 1.14)	1.00	1.28 (0.76, 2.14)	0.90 (0.55, 1.48)	1.27 (0.74, 2.16)	1.06 (0.58, 1.93)	0.84
^c^MV-Adj 3 HR (95% CI)	1.02 (0.87, 1.19)	1.00	1.35 (0.79, 2.31)	0.98 (0.58, 1.68)	1.39 (0.76, 2.52)	1.16 (0.57, 2.35)	0.70

	ACS Score						
	Per point	0–1.5 pts	2–2.5 pts	3–3.5 pts	4–4.5 pts	5–6 pts	*P*_trend_
No. of events/person-years	207/49,493	24/4780	45/9747	60/14,925	43/12,585	35/7457	
Event rate per 1000 person-years	4.2	5.0	4.6	4.0	3.4	4.7	
Age-Adj HR (95% CI)	0.94 (0.84, 1.06)	1.00	0.91 (0.55, 1.51)	0.74 (0.46, 1.20)	0.65 (0.39, 1.07)	0.89 (0.53, 1.51)	0.30
^f^MV-Adj 1 HR (95% CI)	0.93 (0.82, 1.05)	1.00	0.91 (0.55, 1.50)	0.74 (0.46, 1.19)	0.64 (0.39, 1.07)	0.85 (0.50, 1.46)	0.23
^b^MV-Adj 2 HR (95% CI)	0.98 (0.87, 1.11)	1.00	1.09 (0.65, 1.82)	0.89 (0.54, 1.46)	0.87 (0.51, 1.46)	1.05 (0.60, 1.83)	0.75
^c^MV-Adj 3 HR (95% CI)	1.06 (0.91, 1.23)	1.00	1.18 (0.69, 2.01)	1.01 (0.58, 1.75)	1.08 (0.59, 1.96)	1.42 (0.74, 2.73)	0.46

*ACS* American Cancer Society, *AICR* American Institute for Cancer Research, *CI* confidence interval, *HR* hazard ratio, *MV-Adj* multivariable-adjusted, *PCa* prostate cancer, *pts* points, *WCRF* World Cancer Research Fund.

^a^Additionally adjusted for non-Hispanic White race/ethnicity, family history of PCa, selenium, calories, whole milk, wine, processed meat, tomatoes, and fatty fish.

^b^Adjusted for all variables in MV-Adj 1, clinical stage, Gleason score, prostate-specific antigen levels, and treatment.

^c^Adjusted for all variables in MV-Adj 2 and health behaviour score 8–10 years pre-diagnosis.

^d^Additionally adjusted for non-Hispanic White race/ethnicity, family history of PCa, selenium, calories, whole milk, and wine.

^e^Additionally adjusted for non-Hispanic White race/ethnicity, family history of PCa, selenium, calories, smoking, whole milk, and fatty fish.

^f^Additionally adjusted for non-Hispanic White race/ethnicity, family history of PCa, selenium, calories, smoking, whole milk, alcohol, and fatty fish.

Primary analyses of the 2015 PCa Behaviour Score were based on 4320 men, 183 fatal events, and a median follow-up of 8.2 (5.3, 13) years. The score trended significantly inversely associated with the risk of fatal PCa (HR_5–6 pts vs. 0–1 pts_: 0.68, 95% CI: 0.29–1.57, *P*_trend_: 0.02) (Table 3), though event numbers were limited in extreme categories of the score. The absolute crude rates of fatal PCa per 1000 person-years were 3.9 versus 6.5 for those with 5–6 points versus 0–1 points, respectively, and each one-point increase in the score was associated with a 19% reduced risk. Results for the 2015 PCa Behaviour Score Excluding Smoking were attenuated (HR_4–5 pts vs. 0–1 pts_: 0.83, 95% CI: 0.46–1.47, *P*_trend_: 0.07) (Supplementary Table 2).

Primary analyses of the WCRF/AICR Score were based on 4518 men and 208 fatal events, and those for the ACS Score were based on 4505 men and 207 fatal events. Both analyses included a median follow-up of 8.7 (5.7, 14) years. Neither score demonstrated evidence of an association with fatal PCa (Table 3), nor did the ACS Score Including Alcohol (Supplementary Table 2).

We did not observe effect modification by age or stage at diagnosis (*P* > 0.05; data not shown) for any of the primary scores, with the exception of a significant interaction between the WCRF/AICR Score and age at diagnosis (*P*: 0.005). In men younger than 65 years at diagnosis, categorical analyses included insufficient events in the highest category of the WCRF/AICR Score to achieve meaningful estimates, but results trended more strongly inverse in the younger age group (HR_continuous_: 0.59, 95% CI: 0.35–1.00) than the older age group (HR_continuous_: 1.09, 95% CI: 0.92-1.29).

Sensitivity analyses based on simple updating (rather than lagged) exposures yielded statistically significant inverse associations for the 2021 PCa Behaviour Score (HR_3 pts vs. 0–1 pts_: 0.41, 95% CI: 0.26–0.65, *P*_trend_ < 0.001), 2015 PCa Behaviour Score (HR_5–6 pts vs. 0–1 pts_: 0.68, 95% CI: 0.34–1.36, *P*_trend_: 0.01), and ACS Score (HR_5–6 pts vs. 0–1.5 pts_: 0.57, 95% CI: 0.34–0.96, *P*_trend_: 0.02), and a suggestive inverse association for the WCRF/AICR Score (HR_5.25–7 pts vs. 0–2.5 pts_: 0.71, 95% CI: 0.44–1.15, *P*_trend_: 0.13) (Supplementary Table 3). Sensitivity analyses anchoring exposure at first available post-diagnostic questionnaire without updating were similar to those from the primary analyses (Supplementary Table 4). Four to six year lagged analyses including only post-diagnostic score values (versus allowing pre-diagnostic exposures in the lagged exposure definitions) yielded statistically inverse associations of the 2021 PCa Behaviour Score (HR_3 pts vs. 0–1 pts_: 0.48, 95% CI: 0.29–0.81, *P*_trend_: 0.01) and 2015 PCa Behaviour Score (HR_5–6 pts vs. 0–1 pts_: 0.55, 95% CI: 0.26–1.15, *P*_trend_: 0.003) with fatal PCa (Supplementary Table 5). Results for the WCRF/AICR and ACS Scores remained null.

## Discussion

We examined multiple health behaviour scores after diagnosis of PCa for associations with fatal outcomes. In this population of men diagnosed with nonmetastatic PCa, each one-point increase toward not smoking, maintaining a healthy body size, engaging in regular physical activity, and consuming specific food items (2015 PCa Behaviour Score) was associated with a 19% lower risk of dying from PCa. Other scores did not demonstrate convincing evidence of associations with fatal PCa.

Results for the PCa-specific scores were generally attenuated when adjusted for clinical characteristics (i.e., “MV-Adj 2” models), whereas additional adjustment for the relevant pre-diagnostic health behaviour score rendered little impact on association estimates. It could be that current health behaviours are more important than past behaviours when considering PCa survival in men already diagnosed with PCa. That unlagged analyses demonstrated stronger associations between the scores and fatal PCa than lagged analyses supports this possibility.

Among the scores evaluated, the 2015 PCa Behaviour Score demonstrated the strongest inverse relationship with fatal PCa. In contrast to the 2021 PCa Behaviour Score that included total physical activity, the 2015 PCa Behaviour Score was operationalized based on vigorous physical activity and brisk walking. The latter also included three dietary factors (processed meat, tomatoes, and fatty fish) that the former did not. Interestingly, the inclusion of processed meat, saturated fat, whole milk, and wine intakes in the 2021 PCa Behaviour Score Including Diet yielded results similar to the score without diet. These results suggest that: (1) dietary factors may contribute less to risk reduction than the combined effects of not smoking, exercise, and body size; and (2) there may be very specific dietary factors or patterns associated with PCa mortality. Unfortunately, there are few studies with data on post-diagnostic diet in men with PCa, and it should be noted that the 2015 PCa Behaviour Score was developed in the HPFS [7]. While it remains possible that aspects of diet not studied here could be important, the overall dietary patterns captured by the WCRF/AICR and ACS Scores were not strongly associated with PCa outcomes in this population. That said, a diet that helps men maintain a healthy body weight and physical activity seems to be important.

Timing of healthy behaviours also seems to be important. Whereas analyses relating the 2021 and 2015 PCa Behaviour Scores to fatal events in the same 2-year period yielded strongly inverse signals (Supplementary Table 4), lagged analyses of 4–6 years produced attenuated results (Table 3). Were worsening PCa to result in less healthy behaviours, the result would be an inverse relationship between the scores and fatal PCa. That the lagged analyses were attenuated could suggest such reverse causation. Alternatively, it could be that the relevant aetiologic period for exposure is close in time to potential outcomes. Then, we would not expect to see a relationship when evaluating an exposure window occurring years prior to a fatal event.

The WCRF/AICR and ACS Scores excluded smoking habits, not because smoking was deemed unimportant, but rather because both sets of recommendations focus on diet and exercise; both organisations acknowledge the importance of tobacco avoidance for cancer prevention [64–66]. Regardless, the exclusion of smoking from the PCa-specific behaviour scores did not noticeably alter associations with fatal PCa in our data. This may be due to the relatively small number of recent smokers in the HPFS, such that the smoking component of a behaviour score could prove more important in populations with heavier smoking behaviour. Our observation of no associations of the WCRF/AICR and ACS Scores with fatal PCa could also be attributable to the inclusion of dietary factors that are not associated with PCa outcomes. The more relative points allocated to diet appeared to reduce the strength of associations with the risk of fatal PCa. For example, with regard to health outcomes, assigning equal weight in the score to normal BMI category and to zero intake of sugar-sweetened beverages may not be appropriate.

Our population of predominantly White men may limit the generalisability of our findings. Though Black individuals bear a disproportionate burden of PCa incidence and mortality, survivorship research has been primarily conducted in White populations [7]. A recent review from our group [7] found that among 33 recently published studies on post-diagnostic modifiable risk factors, only six included ≥10% Black men [67–72]. Among them, only two, both addressing BMI, reported results stratified by race [67, 68]. One found null relationships in both Black and White men [67], and the other demonstrated a positive relationship among White men only [68]. Studies examining post-diagnostic modifiable risk factors in other racial/ethnic groups underrepresented in research are similarly lacking.

Our study had some additional limitations worth noting. Because health behaviours were self-reported, the exposures may have been moderately misclassified, albeit not with respect to outcomes given the prospective design. Self-reported BMI [73], physical activity [14], and FFQ component foods [15, 16] have been validated in the HPFS. The 2015 PCa Behaviour Score was developed in the full cohort of healthy HPFS men with the outcome of incident lethal PCa [3], and the literature that drove the selection of factors for the 2021 PCa Behaviour Score came from a limited number of study populations, including the HPFS. Some aspects of our models may thus have been overfit, particularly for the 2015 PCa Behaviour Score. To minimise overfitting for the 2021 PCa Behaviour Score, we intentionally included total rather than vigorous physical activity, given that the latter is strongly inversely associated with fatal PCa in the HPFS. Moreover, associations of smoking, BMI, and physical activity with PCa outcomes have been identified in many other cohorts [7]. Nevertheless, our main findings and different intensities of physical activity should be evaluated in other populations, both for validation in similar independent populations and for examination of our findings in Black men, who bear the greatest burden of PCa in the United States. Lastly, there is the possibility of residual confounding in any observational study, though we adjusted for clinical and lifestyle factors, including pre-diagnostic health behaviours. Strengths of this study include repeated measures of smoking habits, weight, physical activity, and diet over time; long follow-up; and ability to evaluate PCa death as an outcome.

In summary, among men with nonmetastatic PCa, a PCa-specific health behaviour score was associated with a 19% lower risk of fatal PCa per one-point increase. For men diagnosed with nonmetastatic PCa, adhering to recommendations to avoid tobacco, maintain healthy body size, and engage in regular physical activity may decrease the risk of dying from PCa. Further research is needed to understand these results in the context of prior associations between individual behavioural risk factors and fatal PCa and to inform tailored cancer survivorship recommendations.

## Supplementary information

Supplementary Tables 1-6

## Data Availability

Data from the Health Professionals Follow-up Study are available via application (https://sites.sph.harvard.edu/hpfs/for-collaborators/).
