# Supplementary material for: Post-diagnostic health behaviour scores in relation to fatal prostate cancer
Source: Br J Cancer. 2022 Aug 26;127(9):1670–9. doi: 10.1038/s41416-022-01948-7 (PMC9596495; doi:10.1038/s41416-022-01948-7)
Supplement: Supplementary file 1 — Supplementary Tables 1-6 [file 41416_2022_1948_MOESM1_ESM.pdf]

Supplementary Table 1. Individual physical activity and food and beverage items included in the sub-components of the 2021 PCa Behavior, 2015 PCa Behavior, WCRF/AICR, and ACS Scores

Sub-Component	Items
**Physical Activity**	
Total	Walking, jogging, running, bicycling, swimming, tennis, squash or racquetball, calisthenics or rowing, other aerobic exercise, low intensity exercise, heavy outdoor work, moderate outdoor work, weight training for arms, weight training for legs, stairs
Vigorous / Vigorous Aerobic	Walking at a very brisk/striding pace, jogging, running, bicycling at a medium or high intensity, swimming at a medium or high intensity, tennis at a medium or high intensity, squash or racquetball, calisthenics or rowing, other aerobic exercise
Moderate	Walking at a normal or brisk pace, bicycling at a low intensity, swimming at a low intensity, tennis at a low intensity, low intensity exercise, heavy outdoor work, moderate outdoor work, weight training for arms, weight training for legs
Moderate Aerobic	Walking at a normal or brisk pace, bicycling at a low intensity, swimming at a low intensity, tennis at a low intensity, low intensity exercise, heavy outdoor work, moderate outdoor work
Brisk Walking	Walking at a brisk or very brisk / striding pace
Strength Training	Weight training for arms, weight training for legs
**Foods and Beverages**	
Wine	Red wine, white wine
Alcohol	Red wine, white wine, regular beer, lite beer, liquor
Processed Meat	Bacon, beef or pork hot dogs, salami or bologna or other processed meat sandwiches, other processed meats (e.g., sausage, kielbasa, etc.), chicken or turkey hot dogs or sausage
Processed Red Meat	Bacon, beef or pork hot dogs, salami or bologna or other processed meat sandwiches, other processed meats (e.g., sausage, kielbasa, etc.)
Red Meat	Regular hamburgers, lean or extra lean hamburgers, beef or lamb as a main dish, pork as a main dish, beef or lamb or pork as a sandwich or mixed dish
Whole Grains[a]	Cold breakfast cereal, cooked oatmeal or cooked oat bran, other cooked breakfast cereal, rye or pumpernickel bread, whole wheat or oatmeal or other whole grain bread, whole grain or whole wheat crackers, other crackers, bagels or English muffins or rolls, muffins or biscuits, brown rice, pasta, other grains (e.g., bulgar, kasha, couscous, etc.), tortillas, potato chips or corn/tortilla chips, pizza, fat free or light popcorn, regular popcorn, breakfast bars, oat bran, wheat germ, Metamucil
Refined Grains[ab]	Frozen yogurt or sherbet or sorbet or low-fat ice cream, artificially sweetened yogurt, breaded fish cakes or pieces or fish sticks, cold breakfast cereal, cooked oatmeal or cooked oat bran, other cooked breakfast cereal, white bread (including pita), rye or pumpernickel bread, whole wheat or oatmeal or other whole grain bread, whole grain or whole wheat crackers, other crackers, bagels or English muffins or rolls, muffins or biscuits, pancakes or waffles, white rice, pasta, tortillas, potato chips or corn/tortilla chips, pizza, home baked cookies, fat free or reduced fat ready-made cookies, other ready-made cookies, doughnuts, home baked cake, ready-made cake, home baked pie, ready-made pie, home baked sweet roll or coffee cake or other pastry, fat free or reduced fat sweet roll or coffee cake or other pastry, other ready-made sweet roll or coffee cake or other pastry, brownies, pretzels, home-made soup without bouillon, chowder or cream soup
Tomatoes	Tomatoes, tomato juice, tomato sauce, tomato soup, salsa, pizza
Whole Fruits and Vegetables (WCRF/AICR)	Raisins or grapes, prunes or dried plums, bananas, cantaloupe, watermelon, avocado, apples or pears, applesauce, oranges, tangerines or clementines or mandarin oranges, grapefruit, strawberries, blueberries, peaches or plums, apricots, mixed dried fruit, tomatoes, tomato juice or V-8, tomato sauce, tomato soup, salsa, mushrooms, celery, string beans, broccoli, cauliflower, cabbage or coleslaw or sauerkraut, Brussels sprouts, raw carrots, cooked carrots, mixed or stir fry vegetables or soup, yellow or orange (winter) squash, eggplant or zucchini or other summer squash, kale or mustard greens or chard, raw spinach, cooked spinach, iceberg or head lettuce, romaine or leaf lettuce, alfalfa sprouts, green or yellow or red pepper, onion as a vegetable or rings or soup, beets

Sub-Component	Items
Whole Fruits and Vegetables (ACS)[c]	Raisins or grapes, prunes or dried plums, bananas, cantaloupe, watermelon, avocado, apples or pears, applesauce, oranges, tangerines or clementines or mandarin oranges, grapefruit, strawberries, blueberries, peaches or plums, apricots, mixed dried fruit, tomatoes, tomato sauce, tomato soup, salsa, mushrooms, celery, string beans, broccoli, cauliflower, cabbage or coleslaw or sauerkraut, Brussels sprouts, raw carrots, cooked carrots, mixed or stir fry vegetables or soup, yellow or orange (winter) squash, eggplant or zucchini or other summer squash, kale or mustard greens or chard, raw spinach, cooked spinach, iceberg or head lettuce, romaine or leaf lettuce, alfalfa sprouts, green or yellow or red pepper, onion as a vegetable or rings or soup, onion as a garnish, beets, yams or sweet potatoes, corn, peas or lima beans fresh or frozen or canned or soup, beans or lentils baked or dried or soup, tofu or soy burger or soybeans or miso or other soy protein, hummus
Fatty Fish	Dark meat fish, e.g., tuna steak, mackerel, salmon, sardines, bluefish, swordfish
Sugar-Sweetened Beverages	Prune juice, apple juice or cider, orange juice, grapefruit juice, other fruit juices, Coke or Pepsi or other cola with sugar, other carbonated beverages with sugar, other sugared beverages (punch, lemonade, sports drinks, or sugared ice tea)
aUPFs	Non-dairy coffee whitener, frozen yogurt or sherbet or sorbet or low-fat ice cream, regular ice cream, sour cream, plain or artificially sweetened yogurt, other flavored yogurt, margarine, “Spreadable Butter,” cottage or ricotta cheese, cream cheese, other cheese, breaded fish cakes or pieces or fish sticks, chicken or turkey sandwich or frozen dinner, refined cold breakfast cereal, white bread (including pita), bagels or English muffins or rolls, crackers, white rice, pasta, muffins or biscuits, pancakes or waffles, French fries, potato chips or corn/tortilla chips, pizza, corn or flour tortillas, low-calorie cola with caffeine, low-calorie caffeine-free cola, other low-calorie beverage with caffeine, other low-calorie beverage without caffeine, dairy coffee drinks, diet nutrition drinks, milk chocolate, dark chocolate, candy bars, candy without chocolate, doughnuts, home baked cake, ready-made cake, home baked pie, ready-made pie, home baked cookies, fat free or reduced fat ready-made cookies, other ready-made cookies, home baked sweet roll or coffee cake or other pastry, fat free or reduced fat sweet roll or coffee cake or other pastry, other ready-made sweet roll or coffee cake or other pastry, brownies, breakfast bars, energy bars, high protein bars, pretzels, fat free or light popcorn, regular popcorn, home-made soup with bouillon, ready-made soup from a can, chowder or cream soup, jams or jellies or preserves or syrup or honey, ketchup or red chili sauce, salad dressing, low-fat or fat-free mayonnaise, regular mayonnaise, artificial sweetener

Abbreviations: ACS – American Cancer Society; AICR – American Institute for Cancer Research; aUPF – adapted ultra-processed food; PCa – prostate cancer; WCRF – World Cancer Research Fund

[a] Grams of grains consumed were derived from raw data on food frequency questionnaires, with each food item contributing a percentage of the total; items listed made up ≥1% of total grams consumed for at least one food frequency questionnaire

[b] Added to whole grains in the denominator of percentage of grains that are whole

[c] When considering variety of whole fruits and vegetables consumed, each of the following sets contributed only one point to variety, as appropriate: apples or pears and applesauce; tomatoes and tomato sauce and tomato soup and salsa; raw carrots and cooked carrots; raw spinach and cooked spinach; onions as a vegetable and onions as a garnish; beans or lentils and hummus

Supplementary Table 2. Secondary health behavior scores lagged by four to six years in relation to risk of fatal PCa for Health Professionals Follow-up Study participants diagnosed with non-metastatic PCa, with follow-up starting at the time of diagnosis

	**2021 PCa Behavior Score Including Diet**						
	Per Point	0-1.5 pts	1.75-2.25 pts	2.5-3 pts	3.25-3.5 pts	3.75-4 pts	P_{trend}
No. of Events/Person-years	182/45,921	16/2433	40/9485	56/15,585	41/11,268	29/7149	
Event Rate Per 1000 Person-years	4.0	6.6	4.2	3.6	3.6	4.1	
Age-Adj HR (95% CI)	0.89 (0.78, 1.02)	1.00	0.51 (0.28, 0.92)	0.42 (0.24, 0.74)	0.44 (0.24, 0.78)	0.46 (0.25, 0.85)	0.05
[a]MV-Adj 1 HR (95% CI)	0.88 (0.77, 1.00)	1.00	0.49 (0.27, 0.89)	0.40 (0.23, 0.71)	0.42 (0.23, 0.75)	0.42 (0.23, 0.79)	0.03
[b]MV-Adj 2 HR (95% CI)	0.94 (0.82, 1.07)	1.00	0.59 (0.33, 1.07)	0.50 (0.28, 0.90)	0.57 (0.31, 1.04)	0.57 (0.30, 1.08)	0.23
[c]MV-Adj 3 HR (95% CI)	1.00 (0.85, 1.17)	1.00	0.64 (0.34, 1.21)	0.57 (0.30, 1.09)	0.68 (0.34, 1.36)	0.75 (0.35, 1.59)	0.79

	**2021 PCa Behavior Score Excluding Smoking Status**						
	Per Point	0 pts	0.5 pts	1 pt	1.5 pts	2 pts	P_{trend}
No. of Events/Person-years	250/56,147	17/2612	40/8906	67/12,195	63/17,768	63/14,666	
Event Rate Per 1000 Person-years	4.5	6.5	4.5	5.5	3.6	4.3	
Age-Adj HR (95% CI)	0.92 (0.83, 1.02)	1.00	0.66 (0.37, 1.16)	0.73 (0.42, 1.24)	0.56 (0.33, 0.96)	0.62 (0.36, 1.06)	0.12
[e]MV-Adj 1 HR (95% CI)	0.92 (0.83, 1.03)	1.00	0.66 (0.37, 1.17)	0.73 (0.42, 1.25)	0.58 (0.33, 0.99)	0.62 (0.36, 1.08)	0.14
[b]MV-Adj 2 HR (95% CI)	0.98 (0.88, 1.10)	1.00	0.68 (0.38, 1.21)	0.86 (0.49, 1.48)	0.68 (0.39, 1.19)	0.79 (0.45, 1.39)	0.74
[c]MV-Adj 3 HR (95% CI)	1.00 (0.88, 1.13)	1.00	0.68 (0.36, 1.27)	0.89 (0.48, 1.63)	0.69 (0.37, 1.30)	0.85 (0.44, 1.63)	0.96

	**2015 PCa Behavior Score Excluding Smoking Status**					
	Per Point	0-1 pts	2 pts	3 pts	4-5 pts	P_{trend}
No. of Events/Person-years	207/49,481	51/9062	95/20,526	37/14,063	24/5831	
Event Rate Per 1000 Person-years	4.2	5.6	4.6	2.6	4.1	
Age-Adj HR (95% CI)	0.79 (0.68, 0.93)	1.00	0.77 (0.55, 1.09)	0.44 (0.29, 0.67)	0.71 (0.44, 1.16)	0.004
[f]MV-Adj 1 HR (95% CI)	0.80 (0.68, 0.94)	1.00	0.79 (0.56, 1.12)	0.45 (0.29, 0.69)	0.74 (0.45, 1.22)	0.007
[b]MV-Adj 2 HR (95% CI)	0.85 (0.72, 1.00)	1.00	0.83 (0.58, 1.19)	0.51 (0.33, 0.78)	0.85 (0.51, 1.42)	0.04
[c]MV-Adj 3 HR (95% CI)	0.85 (0.70, 1.02)	1.00	0.88 (0.60, 1.28)	0.52 (0.32, 0.83)	0.83 (0.46, 1.47)	0.07

	**ACS Score Including Alcohol**						
	Per Point	0-2.5 pts	3-4 pts	4.5-5.5 pts	6-6.5 pts	7-8 pts	P_{trend}
No. of Events/Person-years	207/49,485	17/3336	69/14,480	64/15,720	31/10,181	26/5767	
Event Rate Per 1000 Person-years	4.2	5.1	4.8	4.1	3.0	4.5	
Age-Adj HR (95% CI)	0.91 (0.80, 1.03)	1.00	0.85 (0.50, 1.44)	0.71 (0.41, 1.22)	0.56 (0.31, 1.01)	0.80 (0.43, 1.48)	0.11
[d]MV-Adj 1 HR (95% CI)	0.89 (0.78, 1.02)	1.00	0.85 (0.50, 1.45)	0.71 (0.41, 1.22)	0.54 (0.30, 0.99)	0.75 (0.40, 1.41)	0.08
[b]MV-Adj 2 HR (95% CI)	0.95 (0.83, 1.08)	1.00	0.91 (0.53, 1.56)	0.80 (0.46, 1.40)	0.68 (0.37, 1.25)	0.90 (0.48, 1.72)	0.37
[c]MV-Adj 3 HR (95% CI)	1.06 (0.90, 1.24)	1.00	1.12 (0.62, 2.00)	1.16 (0.62, 2.15)	1.06 (0.53, 2.13)	1.45 (0.68, 3.11)	0.48

Abbreviations: ACS – American Cancer Society; CI – confidence interval; HR – hazard ratio; MV-Adj – multivariable-adjusted; PCa – prostate cancer; pts – points

[a] Additionally adjusted for non-Hispanic white race/ethnicity, family history of PCa, selenium, calories, tomatoes, and fatty fish
[b] Adjusted for all variables in MV-Adj 1, clinical stage, Gleason score, prostate-specific antigen levels, and treatment
[c] Adjusted for all variables in MV-Adj 2 and health behavior score 8-10 years pre-diagnosis
[d] Additionally adjusted for non-Hispanic white race/ethnicity, family history of PCa, selenium, calories, smoking, whole milk, and fatty fish
[e] Additionally adjusted for non-Hispanic white race/ethnicity, family history of PCa, selenium, calories, smoking, whole milk, wine, processed meat, tomatoes, and fatty fish
[f] Additionally adjusted for non-Hispanic white race/ethnicity, family history of PCa, selenium, calories, smoking, whole milk, and wine

Supplementary Table 3. Unlagged health behavior scores in relation to risk of fatal PCa for Health Professionals Follow-up Study participants diagnosed with non-metastatic PCa, with follow-up starting at the time of diagnosis

				**2021 PCa Behavior Score**			
	Per Point	0-1 pts	1.5 pts	2 pts	2.5 pts	3 pts	P_{trend}
No. of Events/Person-years	333/65,281	47/4680	73/10,647	107/15,736	51/18,634	55/15,584	
Event Rate Per 1000 Person-years	5.1	10.0	6.9	6.8	2.7	3.5	
Age-Adj HR (95% CI)	0.76 (0.69, 0.83)	1.00	0.59 (0.40, 0.85)	0.54 (0.38, 0.77)	0.29 (0.19, 0.43)	0.34 (0.23, 0.51)	<0.001
[a]MV-Adj 1 HR (95% CI)	0.75 (0.68, 0.82)	1.00	0.59 (0.41, 0.86)	0.53 (0.38, 0.76)	0.28 (0.19, 0.42)	0.33 (0.22, 0.49)	<0.001
[b]MV-Adj 2 HR (95% CI)	0.81 (0.74, 0.89)	1.00	0.75 (0.51, 1.10)	0.72 (0.50, 1.04)	0.40 (0.27, 0.61)	0.47 (0.31, 0.71)	<0.001
[c]MV-Adj 3 HR (95% CI)	0.78 (0.71, 0.87)	1.00	0.70 (0.47, 1.03)	0.65 (0.44, 0.95)	0.36 (0.23, 0.56)	0.41 (0.26, 0.65)	<0.001
				**2015 PCa Behavior Score**			
	Per Point	0-1 pts	2 pts	3 pts	4 pts	5-6 pts	P_{trend}
No. of Events/Person-years	292/56,736	16/2133	67/9781	125/22,684	55/15,595	29/6543	
Event Rate Per 1000 Person-years	5.2	7.5	6.9	5.5	3.5	4.4	
Age-Adj HR (95% CI)	0.81 (0.72, 0.91)	1.00	0.81 (0.47, 1.40)	0.60 (0.35, 1.01)	0.41 (0.23, 0.72)	0.57 (0.31, 1.05)	<0.001
[d]MV-Adj 1 HR (95% CI)	0.80 (0.71, 0.91)	1.00	0.81 (0.47, 1.41)	0.59 (0.35, 1.00)	0.41 (0.23, 0.71)	0.56 (0.30, 1.04)	<0.001
[b]MV-Adj 2 HR (95% CI)	0.87 (0.77, 0.98)	1.00	0.95 (0.54, 1.66)	0.76 (0.44, 1.30)	0.56 (0.31, 0.99)	0.77 (0.41, 1.45)	0.02
[c]MV-Adj 3 HR (95% CI)	0.84 (0.73, 0.96)	1.00	0.96 (0.54, 1.72)	0.77 (0.44, 1.37)	0.54 (0.29, 1.00)	0.68 (0.34, 1.36)	0.01
				**WCRF/AICR Score**			
	Per Point	0-2.5 pts	2.75-3.25 pts	3.5-4.25 pts	4.5-5 pts	5.25-7 pts	P_{trend}
No. of Events/Person-years	332/62,731	68/7859	57/12,732	117/23,525	54/12,186	36/6430	
Event Rate Per 1000 Person-years	5.3	8.7	4.5	5.0	4.4	5.6	
Age-Adj HR (95% CI)	0.90 (0.82, 0.99)	1.00	0.54 (0.38, 0.77)	0.62 (0.46, 0.84)	0.53 (0.37, 0.75)	0.69 (0.46, 1.04)	0.04
[e]MV-Adj 1 HR (95% CI)	0.88 (0.80, 0.97)	1.00	0.55 (0.38, 0.78)	0.61 (0.45, 0.82)	0.50 (0.35, 0.72)	0.63 (0.41, 0.96)	0.01
[b]MV-Adj 2 HR (95% CI)	0.91 (0.82, 1.00)	1.00	0.56 (0.39, 0.80)	0.64 (0.47, 0.88)	0.56 (0.39, 0.82)	0.68 (0.44, 1.05)	0.07
[c]MV-Adj 3 HR (95% CI)	0.91 (0.82, 1.02)	1.00	0.56 (0.39, 0.80)	0.64 (0.46, 0.89)	0.57 (0.38, 0.86)	0.71 (0.44, 1.15)	0.13
				**ACS Score**			
	Per Point	0-1.5 pts	2-2.5 pts	3-3.5 pts	4-4.5 pts	5-6 pts	P_{trend}
No. of Events/Person-years	326/62,375	44/5860	76/12,687	106/19,056	62/15,453	38/9320	
Event Rate Per 1000 Person-years	5.2	7.5	6.0	5.6	4.0	4.1	
Age-Adj HR (95% CI)	0.85 (0.78, 0.94)	1.00	0.71 (0.49, 1.03)	0.66 (0.46, 0.94)	0.51 (0.34, 0.75)	0.54 (0.35, 0.83)	<0.001
[f]MV-Adj 1 HR (95% CI)	0.84 (0.76, 0.92)	1.00	0.69 (0.47, 1.01)	0.64 (0.45, 0.92)	0.48 (0.33, 0.72)	0.49 (0.31, 0.77)	<0.001
[b]MV-Adj 2 HR (95% CI)	0.89 (0.81, 0.98)	1.00	0.79 (0.54, 1.16)	0.80 (0.56, 1.16)	0.62 (0.42, 0.93)	0.62 (0.40, 0.98)	0.02
[c]MV-Adj 3 HR (95% CI)	0.87 (0.78, 0.98)	1.00	0.81 (0.55, 1.19)	0.80 (0.54, 1.19)	0.61 (0.39, 0.95)	0.57 (0.34, 0.96)	0.02

Abbreviations: ACS – American Cancer Society; AICR – American Institute for Cancer Research; CI – confidence interval; HR – hazard ratio; MV-Adj – multivariable-adjusted; PCa – prostate cancer; pts – points; WCRF – World Cancer Research Fund

[a] Additionally adjusted for non-Hispanic white race/ethnicity, family history of PCa, selenium, calories, whole milk, wine, processed meat, tomatoes, and fatty fish
[b] Adjusted for all variables in MV-Adj 1, clinical stage, Gleason score, prostate-specific antigen levels, and treatment
[d] Adjusted for all variables in MV-Adj 2 and health behavior score 4-6 years pre-diagnosis
[d] Additionally adjusted for non-Hispanic white race/ethnicity, family history of PCa, selenium, calories, whole milk, and wine
[e] Additionally adjusted for non-Hispanic white race/ethnicity, family history of PCa, selenium, calories, smoking, whole milk, and fatty fish
[f] Additionally adjusted for non-Hispanic white race/ethnicity, family history of PCa, selenium, calories, smoking, whole milk, alcohol, and fatty fish

Supplementary Table 4. First post-diagnostic health behavior scores (without updating) in relation to risk of fatal PCa for Health Professionals Follow-up Study participants diagnosed with non-metastatic PCa, with follow-up starting at the time of first available post-diagnostic questionnaire with all score components

	2021 PCa Behavior Score						
	Per Point	0-1 pts	1.5 pts	2 pts	2.5 pts	3 pts	P_{trend}
No. of Events/Person-	369/57,100	38/4380	72/9279	86/12,711	84/16,842	89/13,888	
Event Rate Per 1000 Person-years	6.5	8.7	7.8	6.8	5.0	6.4	
Age-Adj HR (95% CI)	0.89 (0.82, 0.96)	1.00	0.79 (0.53, 1.18)	0.67 (0.46, 0.99)	0.52 (0.35, 0.76)	0.64 (0.43, 0.94)	0.005
[a]MV-Adj 1 HR (95% CI)	0.88 (0.81, 0.96)	1.00	0.81 (0.54, 1.21)	0.67 (0.45, 0.98)	0.51 (0.35, 0.76)	0.62 (0.42, 0.93)	0.003
[b]MV-Adj 2 HR (95% CI)	0.94 (0.86, 1.02)	1.00	1.01 (0.67, 1.52)	0.89 (0.60, 1.32)	0.69 (0.46, 1.03)	0.88 (0.59, 1.33)	0.13
[c]MV-Adj 3 HR (95% CI)	0.93 (0.83, 1.03)	1.00	1.07 (0.69, 1.66)	0.91 (0.59, 1.42)	0.70 (0.44, 1.11)	0.90 (0.55, 1.48)	0.16
	2015 PCa Behavior Score						
	Per Point	0-1 pts	2 pts	3 pts	4 pts	5-6 pts	P_{trend}
No. of Events/Person-	328/48,848	14/1716	70/8292	129/17,922	82/14,154	33/6763	
Event Rate Per 1000 Person-years	6.7	8.2	8.4	7.2	5.8	4.9	
Age-Adj HR (95% CI)	0.86 (0.77, 0.95)	1.00	0.85 (0.48, 1.52)	0.74 (0.42, 1.29)	0.60 (0.34, 1.06)	0.56 (0.30, 1.05)	0.005
[d]MV-Adj 1 HR (95% CI)	0.86 (0.77, 0.96)	1.00	0.86 (0.48, 1.53)	0.74 (0.42, 1.29)	0.61 (0.34, 1.08)	0.56 (0.30, 1.05)	0.006
[b]MV-Adj 2 HR (95% CI)	0.88 (0.79, 0.98)	1.00	1.02 (0.57, 1.84)	0.90 (0.51, 1.58)	0.75 (0.42, 1.34)	0.69 (0.36, 1.31)	0.02
[c]MV-Adj 3 HR (95% CI)	0.84 (0.74, 0.96)	1.00	1.25 (0.68, 2.31)	1.11 (0.59, 2.07)	0.82 (0.43, 1.57)	0.68 (0.33, 1.39)	0.01
	WCRF/AICR Score						
	Per Point	0-2.5 pts	2.75-3.25 pts	3.5-4.25 pts	4.5-5 pts	5.25-7 pts	P_{trend}
No. of Events/Person-	369/54,528	46/6319	67/10,080	119/20,010	84/11,512	53/6608	
Event Rate Per 1000 Person-years	6.8	7.3	6.7	6.0	7.3	8.0	
Age-Adj HR (95% CI)	1.00 (0.92, 1.10)	1.00	0.88 (0.60, 1.28)	0.79 (0.56, 1.11)	0.89 (0.62, 1.28)	0.99 (0.67, 1.48)	0.99
[e]MV-Adj 1 HR (95% CI)	1.00 (0.91, 1.09)	1.00	0.88 (0.60, 1.28)	0.78 (0.55, 1.10)	0.88 (0.61, 1.27)	0.97 (0.64, 1.46)	0.88
[b]MV-Adj 2 HR (95% CI)	1.03 (0.94, 1.13)	1.00	0.80 (0.54, 1.17)	0.85 (0.59, 1.20)	0.97 (0.66, 1.41)	1.00 (0.66, 1.51)	0.53
[c]MV-Adj 3 HR (95% CI)	1.05 (0.94, 1.17)	1.00	0.81 (0.55, 1.20)	0.89 (0.61, 1.28)	1.03 (0.68, 1.55)	1.07 (0.66, 1.72)	0.41
	ACS Score						
	Per Point	0-1.5 pts	2-2.5 pts	3-3.5 pts	4-4.5 pts	5-6 pts	P_{trend}
No. of Events/Person-	363/54,200	44/5554	84/10,854	104/16,820	77/13,672	54/7299	
Event Rate Per 1000 Person-years	6.7	7.9	7.7	6.2	5.6	7.4	
Age-Adj HR (95% CI)	0.95 (0.87, 1.04)	1.00	0.94 (0.65, 1.35)	0.76 (0.53, 1.08)	0.68 (0.46, 0.98)	0.97 (0.65, 1.45)	0.24
[f]MV-Adj 1 HR (95% CI)	0.94 (0.86, 1.03)	1.00	0.92 (0.64, 1.33)	0.75 (0.52, 1.07)	0.67 (0.46, 0.98)	0.94 (0.62, 1.41)	0.21
[b]MV-Adj 2 HR (95% CI)	0.99 (0.90, 1.08)	1.00	0.97 (0.66, 1.40)	0.83 (0.57, 1.19)	0.77 (0.52, 1.13)	1.11 (0.73, 1.69)	0.74
[c]MV-Adj 3 HR (95% CI)	0.97 (0.86, 1.09)	1.00	1.02 (0.68, 1.53)	0.85 (0.56, 1.30)	0.76 (0.48, 1.22)	1.07 (0.63, 1.81)	0.61

Abbreviations: ACS – American Cancer Society; AICR – American Institute for Cancer Research; CI – confidence interval; HR – hazard ratio; MV-Adj – multivariable-adjusted; PCa – prostate cancer; pts – points; WCRF – World Cancer Research Fund

[a] Additionally adjusted for non-Hispanic white race/ethnicity, family history of PCa, selenium, calories, whole milk, wine, processed meat, tomatoes, and fatty fish
[b] Adjusted for all variables in MV-Adj 1, clinical stage, Gleason score, prostate-specific antigen levels, and treatment
[c] Adjusted for all variables in MV-Adj 2 and last health behavior score at least four years prior to relevant first post-diagnostic exposure health behavior score
[d] Additionally adjusted for non-Hispanic white race/ethnicity, family history of PCa, selenium, calories, whole milk, and wine
[e] Additionally adjusted for non-Hispanic white race/ethnicity, family history of PCa, selenium, calories, smoking, whole milk, and fatty fish
[f] Additionally adjusted for non-Hispanic white race/ethnicity, family history of PCa, selenium, calories, smoking, whole milk, alcohol, and fatty fish

Supplementary Table 5. Health behavior scores lagged by four to six years in relation to risk of fatal PCa for Health Professionals Follow-up Study participants diagnosed with non-metastatic PCa, with follow-up starting four to six years after the first available post-diagnostic questionnaire with all score components

	**2021 PCa Behavior Score**						
	Per Point	0-1 pts	1.5 pts	2 pts	2.5 pts	3 pts	P_{trend}
No. of Events/Person-	303/38,143	37/2703	51/6190	82/8730	70/11,109	63/9410	
Event Rate Per 1000 Person-years	7.9	13.7	8.2	9.4	6.3	6.7	
Age-Adj HR (95% CI)	0.86 (0.78, 0.94)	1.00	0.54 (0.35, 0.83)	0.60 (0.40, 0.89)	0.45 (0.30, 0.67)	0.47 (0.31, 0.70)	0.001
[a]MV-Adj 1 HR (95% CI)	0.85 (0.77, 0.93)	1.00	0.53 (0.34, 0.82)	0.58 (0.39, 0.86)	0.44 (0.29, 0.65)	0.44 (0.29, 0.68)	<0.001
[b]MV-Adj 2 HR (95% CI)	0.90 (0.82, 0.99)	1.00	0.63 (0.41, 0.97)	0.73 (0.48, 1.09)	0.56 (0.37, 0.85)	0.58 (0.38, 0.89)	0.02
[c]MV-Adj 3 HR (95% CI)	0.87 (0.78, 0.97)	1.00	0.59 (0.37, 0.93)	0.66 (0.42, 1.04)	0.50 (0.31, 0.80)	0.48 (0.29, 0.81)	0.01
	**2015 PCa Behavior Score**						
	Per Point	0-1 pts	2 pts	3 pts	4 pts	5-6 pts	P_{trend}
No. of Events/Person-	252/31,399	12/1033	61/5282	100/12,321	55/8812	24/3951	
Event Rate Per 1000 Person-years	8.0	11.6	11.6	8.1	6.2	6.1	
Age-Adj HR (95% CI)	0.82 (0.72, 0.93)	1.00	0.91 (0.49, 1.70)	0.62 (0.34, 1.13)	0.51 (0.27, 0.96)	0.56 (0.28, 1.12)	0.002
[d]MV-Adj 1 HR (95% CI)	0.81 (0.71, 0.93)	1.00	0.90 (0.48, 1.68)	0.61 (0.33, 1.12)	0.50 (0.26, 0.94)	0.55 (0.27, 1.10)	0.002
[b]MV-Adj 2 HR (95% CI)	0.85 (0.74, 0.96)	1.00	1.09 (0.58, 2.06)	0.75 (0.40, 1.38)	0.66 (0.35, 1.25)	0.67 (0.33, 1.37)	0.01
[c]MV-Adj 3 HR (95% CI)	0.81 (0.70, 0.93)	1.00	1.03 (0.54, 1.96)	0.68 (0.36, 1.28)	0.57 (0.29, 1.11)	0.55 (0.26, 1.15)	0.003
	**WCRF/AICR Score**						
	Per Point	0-2.5 pts	2.75-3.25 pts	3.5-4.25 pts	4.5-5 pts	5.25-7 pts	P_{trend}
No. of Events/Person-	276/34,689	32/4055	65/6716	87/12,968	64/7075	28/3875	
Event Rate Per 1000 Person-years	8.0	7.9	9.7	6.7	9.1	7.2	
Age-Adj HR (95% CI)	0.97 (0.87, 1.07)	1.00	1.22 (0.80, 1.87)	0.86 (0.57, 1.29)	1.10 (0.72, 1.69)	0.91 (0.54, 1.51)	0.62
[e]MV-Adj 1 HR (95% CI)	0.96 (0.86, 1.06)	1.00	1.22 (0.80, 1.88)	0.84 (0.56, 1.27)	1.08 (0.70, 1.67)	0.85 (0.50, 1.44)	0.47
[b]MV-Adj 2 HR (95% CI)	0.99 (0.88, 1.10)	1.00	1.22 (0.79, 1.88)	0.91 (0.60, 1.37)	1.25 (0.80, 1.96)	0.90 (0.53, 1.54)	0.90
[c]MV-Adj 3 HR (95% CI)	0.97 (0.86, 1.10)	1.00	1.17 (0.76, 1.82)	0.85 (0.55, 1.32)	1.16 (0.72, 1.88)	0.86 (0.47, 1.55)	0.77
	**ACS Score**						
	Per Point	0-1.5 pts	2-2.5 pts	3-3.5 pts	4-4.5 pts	5-6 pts	P_{trend}
No. of Events/Person-	275/34,558	26/3093	74/6888	78/10,509	54/8856	43/5213	
Event Rate Per 1000 Person-years	8.0	8.4	10.7	7.4	6.1	8.3	
Age-Adj HR (95% CI)	0.92 (0.83, 1.02)	1.00	1.25 (0.80, 1.97)	0.85 (0.54, 1.33)	0.73 (0.46, 1.17)	1.02 (0.62, 1.67)	0.12
[f]MV-Adj 1 HR (95% CI)	0.92 (0.82, 1.02)	1.00	1.26 (0.80, 1.98)	0.86 (0.55, 1.35)	0.73 (0.45, 1.18)	0.99 (0.60, 1.65)	0.10
[b]MV-Adj 2 HR (95% CI)	0.96 (0.86, 1.07)	1.00	1.41 (0.89, 2.23)	0.97 (0.61, 1.53)	0.90 (0.55, 1.46)	1.22 (0.73, 2.05)	0.45
[c]MV-Adj 3 HR (95% CI)	0.95 (0.83, 1.08)	1.00	1.50 (0.92, 2.42)	0.99 (0.60, 1.64)	0.89 (0.51, 1.55)	1.18 (0.64, 2.16)	0.40

Abbreviations: ACS – American Cancer Society; AICR – American Institute for Cancer Research; CI – confidence interval; HR – hazard ratio; MV-Adj – multivariable-adjusted; PCa – prostate cancer; pts – points; WCRF – World Cancer Research Fund

[a] Additionally adjusted for non-Hispanic white race/ethnicity, family history of PCa, selenium, calories, whole milk, wine, processed meat, tomatoes, and fatty fish

[b] Adjusted for all variables in MV-Adj 1, clinical stage, Gleason score, prostate-specific antigen levels, and treatment

[c] Adjusted for all variables in MV-Adj 2 and last health behavior score at least four years prior to relevant first post-diagnostic exposure health behavior score

[d] Additionally adjusted for non-Hispanic white race/ethnicity, family history of PCa, selenium, calories, whole milk, and wine

[e] Additionally adjusted for non-Hispanic white race/ethnicity, family history of PCa, selenium, calories, smoking, whole milk, and fatty fish

[f] Additionally adjusted for non-Hispanic white race/ethnicity, family history of PCa, selenium, calories, smoking, whole milk, alcohol, and fatty fish

Supplementary Table 6. Age-adjusted health behaviors for Health Professionals Follow-up Study participants with non-metastatic PCa, according to extreme categories of first health behavior scores following diagnosis; populated fields for each score correspond to score components (regular font) or model covariates (italic font)

	**2021 PCa Behavior Score**		**2015 PCa Behavior Score**		**WCRF/AICR Score**		**ACS Score**	
**Sample Size**	n = 4968		n = 4605		n = 5223		n = 5208	
**# Points:**	**0-1**	**3**	**0-1**	**5-6**	**0-2.5**	**5.25-7**	**0-1.5**	**5-6**
n[a]	423	1170	173	645	635	609	568	705
Smoking Status								
Never	21	56	19	50	*41*	*62*	*40*	*60*
Past – Quit ≥10 Years Prior	31	44	22	49	*46*	*34*	*47*	*36*
Past – Quit <10 Year Prior	19	0.0	40	0.77	*8.0*	*2.8*	*8.2*	*3.1*
Current	30	0.0	19	0.0	*4.6*	*1.2*	*4.4*	*0.44*
Mean Body Mass Index, kg/m^2 ± SD	30 ± 4.5	23 ± 1.3	30 ± 4.8	25 ± 2.5	29 ± 4.3	24 ± 2.3	31 ± 3.9	23 ± 1.7
Mean Physical Activity ± SD								
Total, MET-hr/week	3.8 ± 7.0	54 ± 34	-	-	-	-	-	-
Vigorous, hr/week	-	-	0.34 ± 0.68	4.3 ± 5.2	-	-	-	-
Brisk Walking, hr/wk	-	-	0.25 ± 1.1	3.1 ± 4.7	-	-	-	-
Moderate-Vigorous, hr/wk[b]	-	-	-	-	3.5 ± 5.9	12 ± 10	-	-
Moderate-Vigorous Aerobic, MET-hr/wk	-	-	-	-	-	-	8.8 ± 14	57 ± 42
Strength Training, min/wk	-	-	-	-	-	-	2.1 ± 12	89 ± 128
Supplemental Selenium Use								
Non-User	*92*	*83*	*94*	*79*	*90*	*78*	*92*	*77*
<140 µg/day	*5.3*	*11*	*3.8*	*11*	*6.3*	*13*	*5.1*	*13*
≥140 µg/day	*3.2*	*5.9*	*2.2*	*9.9*	*3.8*	*9.0*	*3.2*	*9.9*
Mean Nutrient and Food Intakes ± SD								
Total Calories, kcal/day	*1915 ± 614*	*2052 ± 612*	*1994 ± 643*	*2221 ± 629*	*2077 ± 657*	*1850 ± 544*	*1972 ± 661*	*2068 ± 595*
Whole Milk, serv/wk	*0.69 ± 2.1*	*0.39 ± 1.8*	*0.84 ± 2.5*	*0.21 ± 0.76*	*0.84 ± 2.4*	*0.26 ± 1.1*	*0.73 ± 2.3*	*0.22 ± 0.95*
Wine, serv/wk	*1.7 ± 3.2*	*2.4 ± 3.3*	*1.5 ± 2.5*	*3.1 ± 3.5*	-	-	-	-
Total Alcohol, g/day	-	-	-	-	14 ± 17	7.1 ± 10	-	-
Total Alcohol, serv/day	-	-	-	-	-	-	*0.87 ± 1.1*	*0.81 ± 0.82*
Processed Meat, serv/wk[c]	*2.8 ± 2.5*	*1.8 ± 2.2*	4.9 ± 2.6	0.97 ± 1.1	-	-	-	-
Red Meat, g/wk	-	-	-	-	499 ± 308	197 ± 198	-	-
Processed Meat, g/wk	-	-	-	-	150 ± 138	28 ± 78	-	-
Red and Processed Meat, serv/wk	-	-	-	-	-	-	1.2 ± 0.69	0.47 ± 0.44
Percentage of Grains that are Whole	-	-	-	-	-	-	0.29 ± 0.15	0.52 ± 0.14
Tomatoes, serv/wk	*5.1 ± 3.4*	*5.2 ± 2.8*	3.9 ± 2.3	9.8 ± 5.6	-	-	-	-
Total Fruits and Vegetables, g/day	-	-	-	-	314 ± 184	639 ± 285	-	-
Total Fruits and Vegetables, serv/day	-	-	-	-	-	-	4.6 ± 2.5	8.1 ± 3.6
Variety of Fruits and Vegetables, #/mo	-	-	-	-	-	-	20 ± 5.4	25 ± 4.9
Fiber, g/day	-	-	-	-	19 ± 6.6	27 ± 9.2	-	-
Fatty Fish, serv/wk	*0.44 ± 0.62*	*0.56 ± 0.62*	0.17 ± 0.31	1.3 ± 1.1	*0.33 ± 0.40*	*0.65 ± 0.69*	*0.35 ± 0.46*	*0.68 ± 0.67*
Sugar Sweetened Beverages, g/day	-	-	-	-	296 ± 272	135 ± 190	-	-
Percentage of Calories from aUPFs	-	-	-	-	0.40 ± 0.09	0.23 ± 0.08	-	-

Notes: all values calculated based on individuals without missing data; percentages may not add up as expected due to rounding; missing values (-) reflect components that were not included in a given score or included as model covariates for a given score

Abbreviations: ACS – American Cancer Society; AICR –American Institute for Cancer Research; aUPF – adapted ultra-processed food; g – grams; hr – hours; MET – metabolic equivalent of task; min – minutes; mo – month; PCa – prostate cancer; serv – servings; WCRF – World Cancer Research Fund; wk – week

[a] Not adjusted for age

[b] Hours per week of vigorous physical activity were doubled in the calculation for overall hours per week of moderate or vigorous physical activity

[c] The 2015 PCa Behavior Score included processed *red* meat only
